# Social Support and Drug Abstention Motivation among Chinese Male Drug Addicts: A Moderated Mediation Model of Self-Control and Sensation-Seeking

**DOI:** 10.3390/ijerph19106015

**Published:** 2022-05-15

**Authors:** Xizheng Xu, Yunpeng Wu, Senlin Zhou

**Affiliations:** 1 Department of Management, Hunan Police Academy, Changsha 410138, China; zhousenlin@hnpa.edu.cn; 2 School of Teacher Education, Dezhou University, Dezhou 253023, China

**Keywords:** social support, abstention motivation, self-control, sensation-seeking, moderated mediation

## Abstract

The goal of this study is to examine how social support affects Chinese male drug addicts’ abstention motivation. To elucidate the mechanism as well as the boundary condition of the aforesaid influence, self-control and sensation-seeking were induced. Using the questionnaire method, the cross-sectional data were collected from 498 male drug addicts from one hospital and four compulsory isolation drug abstention centers in Central China region. The results indicated that social support has a positive direct and indirect effect on abstention motivation. The indirect influence is that the impact of social support on abstention motivation is mediated by self-control. The direct effect was moderated by sensation-seeking. Specifically, for individuals with low sensation-seeking, social support can significantly increase drug abstinence motivation, but this effect was not significant for those with high sensation-seeking. Theoretical and practical implications of the results were discussed.

## 1. Introduction

Drug addiction is not only an important health problem but also a serious societal problem worldwide, threatening human health and social development globally. It is closely related to lifespan shortening and the accelerated transmission of sexually transmitted diseases such as HIV [1] and is also the antecedent variable of crime [2]. Finding factors that can facilitate the treatment effect of drug addiction is gaining special attention from scholars and practitioners [3,4]. Many studies have shown that abstention motivation, defined as a person who can identify the harmful of drug addiction and take measures to give up, is a key factor in the success of drug treatment [5,6]. Therefore, increasing research has been conducted to find the influencing factors of abstention motivation either from an environmental or personality perspective [7,8,9]. Among environmental factors, social support is a crucial variable [10,11]. In personal traits, self-control and sensation-seeking are usually used to explain an individual’s abstention motivation or behavior [12,13].

Although many studies have investigated the role of social support, self-control, and sensation-seeking on abstention motivation, the joint effect of these factors on abstention motivation is rarely unfolded. According to the view of genetic and environmental interaction on delinquent behavior [14], abstention motivation may be influenced by the interaction between internal and external variables. Moreover, previous studies’ conclusions usually drowned from both females and males. Given that men have higher rates and frequencies of drug use than women and more male drug addicts than females in China, it is essential to explore how the above three variables work together to affect males’ abstention motivation. Therefore, the current study proposed a moderated mediation model to explore the influence mechanism of social support on drug abstinence motivation and its boundary condition.

### 1.1. The Role of Social Support on Abstention Motivation

Self-determination theory (SDT) holds that motivation is a continuum from no motivation to internal motivation [15]. Studies based on SDT found that the motivation continuum can be divided into two dimensions called autonomous motivation and control motivation that have significant predictive power for various outcomes [16]. For the above reason, we adopt the comprehension of motivation based on SDT in the current study. Prior studies, in different cultural contexts, indicated that social support provided by family, friends, and other significant persons or organizations has a positive influence on an individual’s motivation for healthy behavior [17,18]. Functionally, social support refers to the spiritual and material support those individuals get from their social relationships [19]. In operation, social support is the quantitative representation of the social relationship individuals have [20]. In line with SDT, social support can not only meet one’s basic psychological needs for a relationship but also provide a safe environment that facilitates an individual’s sense of autonomy and enhances internal motivation [15]. Furthermore, people who lived in collective culture are more sensitive to social relationships such as family and important others [21]. Thus, this positive effect of social support may play a more significant role in an individual’s positive behavior and abstention motivation because of the high collective culture of China. For the above reason, we concentrate on the role of social support in two dimensions of motivations for drug abstention.

**Hypothesis** **1** **(H1).** *Social support is positively related to the abstention motivation of Chinese male drug addicts.*

### 1.2. The Mediating Role of Self-Control

Self-control is a kind of ability that makes a person take full consideration of the behavioral consequences before acting [22]. There are three study directions of the influences of self-control on an individual’s mental state and behavior [23]. The first direction is the influence of low self-control ability on individuals’ negative outcomes. For example, past research showed that low self-control significantly correlated with drug abuse and is one of the most important characteristics of drug addiction [24,25]. The second direction is the beneficial effect of high self-control on individuals’ positive function. For example, high self-control has a positive correlation with people’s subject well-being, and positive emotions [26,27]. The third direction is the mediating role of self-control on the path of independent variables on one’s mental or behavioral outcomes. Many theories and experimental studies have pointed out that self-control ability is a mediating factor that can explain the influence of environmental factors on one’s mental and behavior, such as internal and external problems [24]. It is reasonable to speculate that social support may influence abstention motivation in the form of an indirect path, and self-control may explain this indirect effect.

**Hypothesis** **2** **(H2).** *Self-control mediates the relationship between social support and abstention motivation of Chinese male drug addicts.*

### 1.3. The Moderating Role of Sensation-Seeking

Sensation-seeking is commonly defined as a relatively stable personality trait, including the search for novel and intense sensations or experiences [28]. Extensive evidence has shown that sensation-seeking was strongly related to drug abuse [29,30]. Different from self-control, sensation-seeking is a more stable trait that cannot be easily changed, and the environmental factors may have a different impact on individuals’ outcomes because of the variation of sensation-seeking. In other words, sensation-seeking can be worked as a moderator. For instance, Wei et al. conducted a questionnaire study on 854 Chinese adolescents. The result illustrated that sensation-seeking moderated the relationship between depression and no suicide self-injury [31]. When it comes to our study, it can be informed that individuals who have a high level of this risky personal trait may find it hard to benefit from social support.

**Hypothesis** **3** **(H3).** *Sensation-seeking moderates the relationship between social support and abstention motivation of Chinese male drug addicts. Specifically, for individuals with low sensation-seeking, social support positively correlates with abstention motivation significantly, but this relationship is not significant for individuals with high sensation-seeking.*

### 1.4. The Present Study

**Figure 1:**
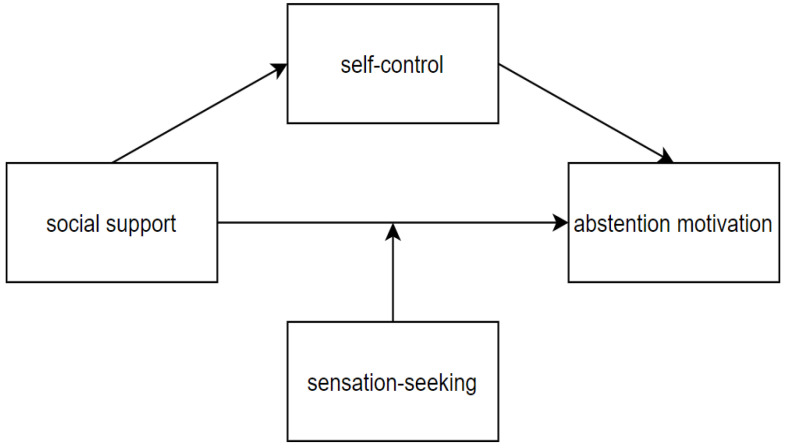
The proposed model.

Based on the above hypotheses, we set up a moderated mediation model to explore three questions. The first question is the role of social support on abstention motivation in the Chinese male population. The second question is the mechanism of the influence of social support on abstention motivation, which is the mediating role of self-control. The third question is about the boundary condition of the above influences, in another word, the moderating role of sensation-seeking. The proposed model is in Figure 1.

## 2. Materials and Methods

### 2.1. Participants

A convenience sampling method was employed to obtain participants on 14 May 2021, from four compulsory isolation drug abstention centers and one hospital in central China. The four compulsory isolation drug abstention centers and one hospital recruit patients from all regions of China. A total of 553 male patients were selected as participants. The participants whose more than 20% data were missing and whose variable scores were more than plus or minus 3 standard deviations were deleted. Finally, 498 valid data were obtained with an effective recovery rate of 90%. The average age of participants is 36.42 years old (range from 18 to 56, SD = 3.51). About 76% of participants received no more than 12 years of school education, 21% got a bachelor’s degree, and 3% got a master’s degree or more. About 28% of them are unemployed, 37% of them have part-time jobs, and 35% of them have stable jobs. About 58% of the participants were heroin users, 28.1% were heroin and other drugs mixed users, 7.6% were meth users, and 6.3% were cocaine users. For the marital status, 53% were married, 27.4% were divorced, and 19.6% of the sample were single. Furthermore, 46% of them were abstinent for less than one year, and 54% of them were abstinent for one to two years. All the participants received the methadone maintenance treatment and patients who received psychotherapy are excluded.

### 2.2. Research Tools

The English version scales used in the study are translated and back-translated to minimize discrepancies between English and Chinese. Specifically, the English versions of scales were translated into Chinese. Then the Chinese versions were back-translated into English. Then translated items were compared and the Chinese versions were revised if the back-translated versions did not match the meaning of the original scales. All the scales have good reliability and Cronbach’s alpha coefficients of the scales are illustrated in Table 1.

#### 2.2.1. Social Support

Social support was measured by the Chinese version of Social Support Scale revised by Xiao [32], which included 10 items and three dimensions, namely, actual objective support, subjective experience of support, and utilization of support. Likert-style four-point scoring was used. The higher the total score, the higher the degree of social support.

#### 2.2.2. Self-Control

We used the Chinese version of Reversed Self-control Scale (RSCS) with a total of 19 items and five dimensions to measure an individual’s score of self-control [33]. The five dimensions of the scale include impulse control, healthy habits, resisting the temptation to Focus on work, entertainment control, and resisting temptation. The RSCS had good construct validates. The total score is used as the general ability of self-control, the higher the total score the more self-control ability.

#### 2.2.3. Sensation-Seeking

Sensation-seeking was measured by the Sensation-seeking Scale developed by Arnett [34], which adopted the Four-point Likert scoring method, including two sub-scales of intensity and novelty. The higher the total score, the higher the trait of sensation-seeking.

#### 2.2.4. Abstention Motivation

This study adjusted the treatment self-regulation questionnaire (TSRQ) developed based on SDT [35], which is usually used to assess the degree of three health behavior scenarios including quitting smoking, healthy eating, and exercise. In our study, we changed the description of these behaviors to drug addiction behavior. The reversed questionnaire aims to assess different forms of abstention motivation to understand why people enter detoxification centers for detoxification activities. One example of an item is “Why do you abstain from the drug? Because stopping the drug is very important for being as healthy as possible”. Seven-point Likert scoring method was used in this scale.

The original scale has 15 items and 4 dimensions, namely, autonomous motivation, introjection regulation, external regulation, and amotivation. Based on the prior study, we combine introjection regulation and external regulation to control motivation and no longer use the amotivation dimension [19,20]. Confirmatory factor analysis showed that the two dimensional (autonomous abstention and control abstention motivation) structure model results were acceptable, with CFI = 0.91, GFI = 0.92, and RMSEA = 0.06.

### 2.3. Procedure

This study was approved by the local ethics committee. We conducted group testing in the local drug abstention centers. The persons in charge of drug abstention centers and the participants provided written consent. All participants were informed that their participation would be voluntary, and they are free to withdraw their participation from this study at any time. Meanwhile, the anonymous nature of the study was emphasized before data collection. Participants were asked to answer independently according to their actual situation. It took about 20 min for the subjects to complete the questionnaire.

### 2.4. Statistical Analysis

R software (version 4.1.3) and “bruceR” (broadly useful convenient and efficient R functions) package (version 0.8.x) were used as statistical tools [36,37]. As the data of all variables in this study were obtained by the self-reporting method, common method bias (CMB) may be brought about [38]. Accordingly, we first conduct Harman single factor test to detect the CMB by EFA (exploratory factor analysis) argument in “bruceR” package. Second, the descriptive statistical magnitudes such as the mean, standard deviation, and Pearson correlation coefficients of interest variables are obtained by “Describe” and “corr” argument in “bruceR” package (see Table 1). Finally, the scores of all variables were standardized, with age and duration of drug use as covariates in the following models. Moderated mediation analysis was performed with “PROCESS” argument which can analyze two regression models based on least-square method. In one model, self-control regresses on social support and covariates. In the other model, autonomous abstention motivation and control abstention motivation respectively regress on social support, self-control, sensation-seeking, and covariates. The bootstrap method was used to sample 5000 times for estimating the confidence intervals of coefficients. In this study, 86.1% of addicts took drugs of heroin or mixed-use heroin and other drugs, so the study did not take the type of drug use as an independent variable.

## 3. Results

The results of our analysis include three parts, namely, the result of the common method bias check; the descriptive statistics and correlation among variables of interest; the regression models including regression coefficients, standard error, and significance sign.

### 3.1. Common Method Bias Test and Covariance

To control the CMB, the method of reverse scoring and appropriate transformation of instruction language measures are taken for the experimental control. Statistically, common method factors accounted for 18% of the total variation, less than the 40% standard, indicating that there was no significant common method bias in the study. There was no significant correlation between the covariance and abstention motivation (*p*s > 0.05), so it will not be discussed in the subsequent analysis.

### 3.2. Descriptive Statistics and Correlation Analysis

Table 1 shows that social support was significantly correlated with self-control and motivation in all dimensions. Self-control was significantly correlated with the two dimensions of abstention motivation. Sensation-seeking was negatively correlated with the two dimensions of abstention motivation significantly.

### 3.3. Results of Regression Models

Results (Table 2) indicated that after controlling other independent variables, social support positively predicted self-control (*b* = 0.14, *p* < 0.01), autonomous abstention motivation (*b* = 0.14, *p* < 0.01)), and control abstention motivation significantly (*b* = 0.13. *p* < 0.01). Hypothesis 1 was supported.

The indirect path coefficients of social support to autonomous and control abstention motivation are significant, which explained 18% and 19% of the total effect of social support on autonomous abstention motivation and control abstention motivation. Meanwhile, there are still direct effects of social support on abstention motivation that cannot be explained by self-control (see Table 2). Hypothesis 2 was supported.

The interaction terms of social support and sensation-seeking had significant effects on the autonomous and control abstention motivation. These results indicate that sensation-seeking has a significant moderating effect on the direct influence of social support on autonomous abstention motivation and control abstention motivation. For the above result, Hypothesis 3 was supported.

**Figure 2:**
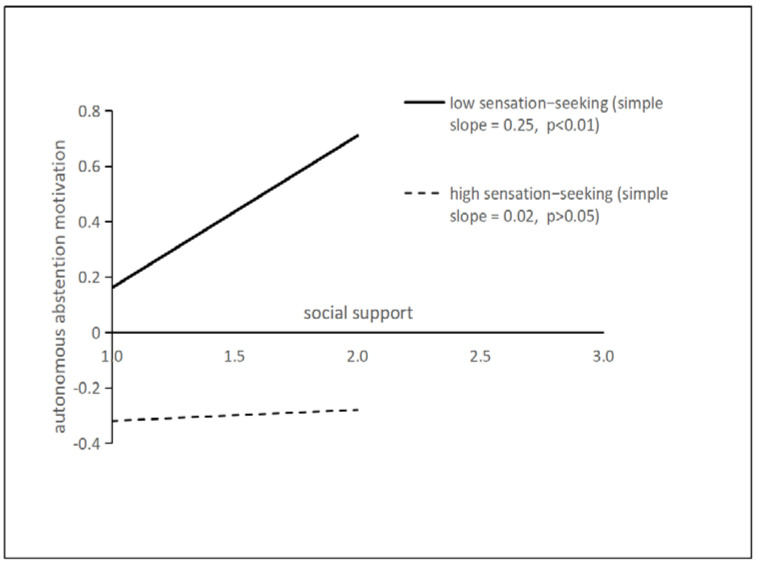
The moderating effect of sensation-seeking on the effect of social support on autonomous abstention motivation.

**Figure 3:**
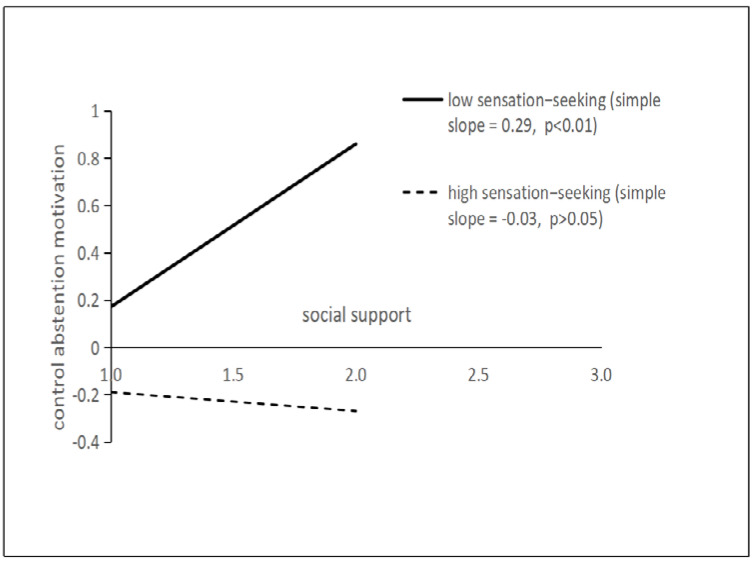
The moderating effect of sensation-seeking on the effect of social support on control abstention motivation.

To reveal the specific mode of the moderating effect more clearly, the analysis charts of the effects of social support on autonomic abstention motivation and control abstention motivation were respectively taken when sensation seeking was plus or minus one standard deviation. As can be seen from Figure 2, social support has a significant promoting effect on autonomous abstention motivation when the trait of sensation-seeking is at a low level (simple slope = 0.25, *p* < 0.01), but when it is at a high level the significant effect disappeared (simple slope = 0.02, *p* > 0.05). From Figure 3, social support has a significant promoting effect on control abstention motivation when the trait of sensation-seeking is at a low level (simple slope = 0.29, *p* < 0.01), but it was not the case when sensation-seeking is at a high level (simple slope = −0.03, *p* > 0.05).

## 4. Discussion

The abstention motivation is affected by both the external environment and individual characteristics. Under the guidance of this view, this study constructed a moderated mediation model in which social support affects drug abstinence motivation through self-control, and the direct effect of social support on drug abstinence motivation is moderated by sensation-seeking traits, which was supported by our results.

Our findings have several contributions. First, the results of our study are like other study conclusions in western cultural backgrounds [39], which expands the effect of social support and sensation-seeking on drug abstention motivation to a broader context. Second, the research findings clarified the influence mechanism of social support on abstention motivation and their boundary condition. Last, it also provided an empirical reference for formulating effective drug abstinence programs for improving abstention motivation in practice.

### 4.1. Social Support and Abstention Motivation

This study found that social support can significantly positively predict abstention motivation, which is consistent with previous studies. The main effect model of social support on mental health believes that social support has a general gain effect on individual mental health [40], and the motivation of drug withdrawal is a positive and healthy psychological process in which individuals establish and strive for the goal of drug withdrawal. The results of this study extend the explanatory scope of the model from the motivation perspective, that is, sufficient social support is significantly positively associated with higher abstinence motivation. The findings show that social support strategies, including the acceptance of emotional support from family members to social institutions and groups, and a series of non-discriminatory policy support made by the government, can improve abstention motivation and further improve the possibility of successful drug abstention.

### 4.2. The Mediating Role of Self-Control

This study further took social support as an antecedent variable and self-control as a mediating variable to test the influence of the two variables on drug abstinence motivation. The study found that social support affected drug abstinence motivation through the mediating effect of self-control, which further verified the mechanism of external environmental factors influencing psychology and behavior through an individual internal cognitive process. Resource theory points out that self-control is a kind of cognitive resource [22]. Individuals use this resource to maintain and adjust to various psychological and behavioral activities when other resources are insufficient or depleted. Our study found that social support can effectively provide resources for individuals and raise positive motivation. The current results have important implications for the practice of drug abstinence, that is, self-control can be improved through social support, and improving self-control in various drug abstinence measures can effectively improve the motivation for drug abstinence.

### 4.3. The Moderating Role of Sensation-Seeking

Previous studies have found that sensation-seeking, as a personality trait of individuals seeking novel experiences, is an important predictor of addictive behavior. This study found that sensation-seeking can also hinder the improvement of abstention motivation. In addition, we also found that sensation-seeking significantly affected the promoting effect of social support on drug abstinence motivation, namely, compared to individuals with high sensation-seeking, social support to individuals with low sensation-seeking could promote the level of abstention motivation more. These results indicate that high sensation-seeking, as a risk factor, can not only directly hinder the promotion of motivation to abstinence but also interact with social support to hinder the motivation by inhibiting the development of self-control. Therefore, in the practice of drug addiction, individuals with high sensation-seeking should be regarded as a special group, and effective measures should be taken to reduce the negative impact of sensation-seeking.

### 4.4. Limitations and Future Research Directions

Our study has some limitations as follows. First, our conclusion is based on cross-sectional data which cannot investigate causal relationships. Second, the data are collected from the attitude questionnaire which may be confounding with social approval. Finally, the participants were recruited from one province in China, and if the conclusion can be generalized to a broader region need further exploration.

Future studies can be conducted by several measures as follows: behavior experimental study should be designed to propose the causal relationship. Longitudinal design should be conducted to detect the dynamic change relationship of variables. electroencephalogram (EEG) and functional magnetic resonance imaging (fMRI) should be induced to find the neuron-basis evidence for these relationships.

## 5. Conclusions

In summary, the current study found that social support can be a beneficial factor for Chinese male addicts’ drug abstinence motivation. Further, self-control played a mediating role in the link between social support and abstinence motivation. Sensation-seeking moderated the effect of social support on drug abstinence motivation. Specifically, for low sensation-seeking individuals, social support may increase their drug abstinence motivation, while this effect was not significant for high sensation-seeking individuals.

The above findings shed light on the practical work of drug abstention in hospitals and drug treatment centers. There are several proposals induced from our findings for increasing social support in drug abstention centers and hospitals. First, doctors nurses, and employees who worked for the drug addicts should receive training courses on getting along with patients and empathy skills to give them affectional support efficiently. Second, the policy supports based on the principle of non-discrimination should be formulated that can make addicts easily go back to society. Third, the treatment program should take addicts’ families and close friends as important resources for treatment. For example, executors can organize family members’ or close friends’ meetings at regular intervals during treatment. However, when it comes to addicts who have a high-level sensation-seeking trait, alternative treatment programs such as physical exercise may be suitable.

## Figures and Tables

**Table 1:** Means, standard deviations, and Pearson correlation coefficients of main variables.

	M	SD	1	2	3	4	5	6	7
1 age	36.42	3.51							
2 drug use time	8.32	1.35	0.69 ***						
3 social support	35.94	8.07	0.07	−0.04	(0.76)				
4 self-control	3.07	0.52	0.08	−0.06	0.17 **	(0.86)			
5 autonomous abstention motivation	5.27	1.15	0.06	0.05	0.17 **	0.22 **	(0.78)		
6 control abstention motivation	5.39	1.23	0.07	0.05	0.16 **	0.23 **	0.62 **	(0.74)	
7 sensation-seeking	3.26	0.89	0.05	0.08	0.06	0.001	−0.10 *	−0.11 *	(0.88)

Note: index in ( ) is the Cronbach’s *a* of the scale; *** *p* < 0.001, ** *p* < 0.01, * *p* < 0.05.

**Table 2:** The regression models of all variables.

Path	*b*	*SE*	Boot 95% *CI*
social support → self-control	0.14 **	0.04	[0.08, 0.25]
social support → autonomous abstention motivation	0.14 **	0.05	[0.06, 0.27]
social support → control abstention motivation	0.13 **	0.03	[0.08, 0.21]
self-control → autonomous abstention motivation	0.19 **	0.04	[0.09, 0.32]
self-control → control abstention motivation	0.21 **	0.06	[0.10, 0.34]
sensation-seeking → autonomous abstention motivation	−0.10 *	0.03	[−0.44, −0.17]
sensation-seeking → control abstention motivation	−0.11 *	0.02	[−0.43, −0.19]
social support × sensation seeking → autonomous abstention motivation	−0.13 **	0.03	[−0.20, −0.06]
social support × sensation seeking → control abstention motivation	−0.16 **	0.04	[−0.23, −0.09]
social support → self-control → autonomous abstention motivation	0.03	-	[0.01, 0.06]
social support → self-control → control abstention motivation	0.03	-	[0.01, 0.06]

Note: The first column is the regression path with independent variables and dependent variables from left to right; *b* is the unstandardized regression coefficient, *SE* is the standard error, boot 95% *CI* is the confidence interval of the standardized regression coefficient obtained by sampling 5000 times with the Bootstrap method. ** *p* < 0.01; * *p* < 0.05.

## Data Availability

The data presented in this study are available on request from the corresponding author. The data are not publicly available due to privacy.

## References

[1] Cho A.K., Melega W.P. (2002). Patterns of methamphetamine abuse and their consequences. J. Addict. Dis..

[2] Petzold J., Szumlinski K.K., London E.D. (2021). Targeting mGlu5 for Methamphetamine Use Disorder. Pharmacol. Ther..

[3] Friedmann P.D., Hendrickson J.C., Gerstein D.R., Zhang Z. (2004). The effect of matching comprehensive services to patients’ needs on drug use improvement in addiction treatment. Addiction.

[4] Lee M.Y., Lee B.H., Kim H.Y., Yang C.H. (2021). Bidirectional role of acupuncture in the treatment of drug addiction. Neurosci. Biobehav. Rev..

[5] Goodman I., Henderson J., Peterson-Badali M., Goldstein A.L. (2015). The relationship between psychosocial features of emerging adulthood and substance use change motivation in youth. J. Subst. Abuse Treat..

[6] Zeng X.Q., Chen Y.L. (2020). Associations of deviant peer affiliation with youths’ substance use disorder abstention motivation: The mediating role of perceived social support and the moderating role of collective identity. J. Ethn. Subst. Abuse..

[7] Kazan Kizilkurt O., Ferzan Gıynaş F. (2019). Factors affecting treatment motivation among Turkish patients receiving inpatient treatment due to alcohol/substance use disorder. J. Ethn. Subst. Abuse..

[8] Testino G., Pellicano R. (2021). Liver stiffness: A motivational tool for achieving alcohol abstention in alcohol use disorder patients?. Minerva Gastroenterol..

[9] Hong P., Li S., Yu Y., Deng Q. (2022). How to enhance the motivation for drug detoxifification: Consciousness guidance and behaviour restriction of family intergenerational Ethics. Int. J. Environ. Res. Public Health.

[10] Cao Q., Liang Y. (2017). Perceived social support and life satisfaction in drug addicts: Self-esteem and loneliness as mediators. J. Health Psychol..

[11] Deng Y., Li X., Liu L., Chui W.H. (2021). Suicide attempts and perceived social support among Chinese drug users: The mediating role of self-esteem and depression. Int. J. Environ. Res. Public Health.

[12] Allahverdipour H., Macintyre R., Hidarnia A., Shafii F., Emami A. (2007). Assessing protective factors against drug abuse among high school students: Self-control and the extended parallel process model. J. Addict. Nurs..

[13] Tomko R.L., Bountress K.E., Gray K.M. (2016). Personalizing substance use treatment based on pre-treatment impulsivity and sensation seeking: A review. Drug Alcohol Depend..

[14] Azeredo A., Moreira D., Figueiredo P., Barbosa F. (2019). Delinquent behavior: Systematic review of genetic and environmental risk factors. Clin. Child. Fam. Psychol. Rev..

[15] Ryan R.M., Deci E.L. (2000). Self-determination theory and the facilitation of intrinsic motivation, social development, and well-being. Am. Psychol..

[16] Vansteenkiste M., Sheldon K.M. (2006). There’s nothing more practical than a good theory: Integrating motivational interviewing and self-determination theory. Br. J. Clin. Psychol..

[17] Chiara G.D., Acquas E., Carboni E. (2010). Drug motivation and abuse: A neurobiological perspective. Ann. N. Y. Acad. Sci..

[18] Uchino B.N., Cacioppo J.T., Kiecolt-Glaser J.K. (1996). The relationship between social support and physiological processes: A review with emphasis on underlying mechanisms and implications for health. Psychol. Bull..

[19] Bedaso A., Adams J., Peng W., Sibbritt D. (2021). The relationship between social support and mental health problems during pregnancy: A systematic review and meta-analysis. Reprod Health.

[20] Kumar N., Oles W., Howell B.A., Janmohamed K., Lee S.T., Funaro M.C., O’Connor P.G., Alexander M. (2021). The role of social network support in treatment outcomes for medication for opioid use disorder: A systematic review. J. Subst. Abuse Treat..

[21] Lu H., Su Y., Wang Q. (2008). Talking about others facilitates ToM in Chinese preschoolers. Dev. Psychol..

[22] Muraven M., Tice D.M., Baumeister R.F. (1998). Self-control as limited resource: Regulatory depletion patterns. J. Pers. Soc. Psychol..

[23] de Ridder D.T.D., Lensvelt-Mulders G., Finkenauer C., Stok F.M., Baumeister R.F. (2012). Taking Stock of Self-Control: A Meta-Analysis of How Trait Self-Control Relates to a Wide Range of Behaviors. Pers. Soc. Psychol. Rev..

[24] Fu K.W., Tremayne K.S. (2021). Self-efficacy and self-control mediate the relationship between negative emotions and attitudes toward plagiarism. J. Acad. Ethics.

[25] Csa B. (2022). Impaired control in addiction involves cognitive distortions and unreliable self-control, not compulsive desires and overwhelmed self-control. Behav. Brain Res..

[26] Hofmann W., Luhmann M., Fisher R.R., Vohs K.D., Baumeister R.F. (2014). Yes, but are they happy? effects of trait self-control on affective well-being and life satisfaction. J. Personal..

[27] Hamama L., Ronen T., Schar K., Rosenbaum M. (2013). Links between stress, positive and negative affect, and life satisfaction among teachers in special education schools. J. Happiness Stud..

[28] Quinn P.D., Harden K.P. (2013). Differential changes in impulsivity and sensation seeking and the escalation of substance use from adolescence to early adulthood. Dev. Psychopathol..

[29] Regan T., Thamotharan S., Hahn H., Bethany H., Solangia E., Jordan S., Sherecce F.A. (2020). Sensation seeking, sexual orientation, and drug abuse symptoms in a community sample of emerging adults. Behav. Pharmacol..

[30] Wasserman A.M., Shaw-Meadow K.J., Moon T.J., Karns-Wright T.E., Mathias C.W., Hill-Kapturczak N., Dougherty D.M. (2021). The externalizing and internalizing pathways to marijuana use initiation: Examining the synergistic effects of impulsiveness and sensation seeking. Dev. Psychol..

[31] Wei C., Li J., Yu C., Chen Y., Zhen S., Zhang W. (2021). Deviant peer affiliation and non-suicidal self-injury among Chinese adolescents: Depression as a mediator and sensation seeking as a moderator. Int. J. Environ. Res. Public Health.

[32] Xiao S.Y. (1994). Theoretical basis, and research application of social support rating scale. J. Clin. Psychiatry.

[33] Tangney J.P., Baumeister R.F., Boone A.L. (2004). High self-control predicts good adjustment, less pathology, better grades, and interpersonal success. J. Personal..

[34] Arnett J. (1994). Sensation seeking: A new conceptualization and a new scale. Pers. Individ. Differ..

[35] Levesque C.S., Williams G.C., Elliot D., Pickering M.A., Bodenhamer B., Finley P.J. (2007). Validating the theoretical structure of the Treatment Self-Regulation Questionnaire (TSRQ) across three different health behaviors. Health Educ. Res..

[36] R Core Team (2016). R: A Language and Environment for Statistical Computing.

[37] Bao H.-W.-S. (2022). bruceR: Broadly Useful Convenient and Efficient R Functions. https://CRAN.R-project.org/package=bruceR.

[38] Siemsen E., Roth A., Oliveira P. (2010). Common Method Bias in Regression Models with Linear, Quadratic, and Interaction Effects. Organ. Res. Methods.

[39] Opara I., Lardier D.T., Fernandez Y., Garcia-Reid P., Reid R.J. (2020). Intrapersonal psychological empowerment profiles on ethnic identity, social support, and lifetime drug use among Hispanic adolescents girls. J. Ethn. Subst. Abuse.

[40] Thoits P.A. (2011). Mechanisms linking social ties and support to physical and mental health. J. Health Soc. Behav..

